# Evaluation of outpatient clindamycin prescriptions across a large military healthcare network

**DOI:** 10.1017/ash.2025.10122

**Published:** 2025-10-27

**Authors:** Mark Derasmo, Kayla Scheps, Joseph E. Marcus

**Affiliations:** 1 Infectious Disease Service, JBSA Fort Sam Houston, Brooke Army Medical Center, TX, USA; 2 Department of Medicine, Uniformed Services University of Health Sciences, Bethesda, MD, USA

## Abstract

**Objective:**

Despite concerns regarding toxicity and antimicrobial resistance, clindamycin prescriptions have remained significant at a large military healthcare system. This study evaluates patient and prescriber factors associated with outpatient clindamycin prescriptions.

**Design and setting:**

This study evaluated clindamycin prescriptions filled between January and December 2023 at outpatient pharmacies in a large military healthcare system.

**Patients:**

During the study period there were 1046 outpatients prescriptions for clindamycin among 972 adult and pediatric patients.

**Results:**

The cohort was predominately female (576, 55.1%) with a median age 48 [IQR 27.5–66]. The clinics with the most prescriptions were the emergency department (45.4%), primary care (23.6%), and surgical clinics (14.9%). While there were 533 prescribers, the ten highest writers of clindamycin accounted for 18.1% of all prescriptions. Beta-lactam allergy (38.5% vs. 16.0%, *p* ≤ 0.00001) was more common in patients with a dental indication and less common in those with a skin and soft tissue infection (51.3% vs. 23.3%, *p* ≤ 0.00001).

**Conclusions:**

Despite local guidelines, clindamycin was still frequently used for a variety of indications in a large military healthcare system with high clindamycin resistance rates. Additionally, a small number of providers were found to be responsible for a disproportionate number of clindamycin prescriptions, highlighting potential targets for intervention for future antimicrobial stewardship interventions.

## Introduction

Clindamycin is a lincosamide antibiotic that targets the 50S ribosome of gram-positive and anaerobic bacteria. Historically, it has been recommended for the treatment of methicillin resistant *Staphylococcus aureus* (MRSA) and for skin and soft tissue infections, especially in patients with known beta-lactam allergies.^1,2^ However, more recently, concerns have been raised regarding increasing resistance rates and potential toxicities.^3–5^ Given its association with *Clostridioides difficile* infection (CDI), reduction of clindamycin prescriptions has been identified as a target in multiple guidelines.^6,7^ Additionally, the American Heart Association has updated their guidance to no longer recommending the use of clindamycin for antibiotic prophylaxis in dental procedures.^8^ While the American Dental Association has clindamycin listed as an alternative agent for dental infections, they provide a very narrow scope of patients who would receive antibiotics.^9^

Various strategies have been employed to reduce clindamycin prescriptions. These efforts have primarily been described in the inpatient setting, and some have shown decreases in CDI.^7^ However, strategies to decrease outpatient antibiotic prescriptions are not as well described. At this large military healthcare system, clindamycin has <60% coverage of MRSA. The antimicrobial stewardship committee’s main strategy to decrease outpatient clindamycin prescribing has been through clinical practice guidelines (CPGs), which do not recommend clindamycin for any outpatient condition. Despite these recommendations, there remain a significant number of clindamycin outpatient prescriptions each year, although rates have been declining since 2018. The driver of these prescriptions is unknown. This study describes prescriber, diagnostic, and patient factors contributing to outpatient clindamycin usage in a large military medical system.

## Methods

### Study design and data collection

This retrospective observational study evaluated outpatient prescriptions of oral clindamycin at all outpatient pharmacies at a large military healthcare system between January 1^st^ and December 31^st^, 2023. This outpatient pharmacy network fills prescriptions from both military clinicians as well as civilian providers in the community for all military service members, retirees, and their family members from both clinics as well as emergency care settings without co-pay. Full dental capabilities with dental subspecialties and postgraduate dental education are provided on base. For military providers, there are CPGs established by the multidisciplinary Antimicrobial Stewardship Committee that recommend against empiric clindamycin in our healthcare system due to high resistance rates in *Staphylococcus aureus*; however, infectious diseases approval is not required to start therapy. On the contrary, novel tetracyclines and linezolid are currently on the restricted formulary and require infectious diseases approval for prescription.

For each prescription filled, duration of therapy and prescribing clinic were collected by chart review. Providers were also reviewed to determine individual prescribing patterns. Dental indications included both prophylaxis for dental procedures as well as treating active dental infections, as many prescriptions were written from outside the healthcare system without ability to determine full indication. On the contrary, for all non-dental procedures, the indications evaluated were based on presence of an infection or alternatively for prophylaxis. When an indication could not be identified from clinical records, its indications was listed as unknown. Patient demographic information including age, gender, military affiliation, and antibiotic allergies was also collected.

### Statistical analysis

Descriptive statistics were performed using percentages for categorical data and medians and interquartile ranges for continuous data, as appropriate. χ^2^ testing was used to compare differences in the numbers of patients with and without known beta-lactam allergies for each indication. *P*-values of <0.05 were predetermined to be statistically significant.

This protocol was reviewed by the San Antonio Institutional Review Board and it was determined to be a quality improvement project of the antimicrobial stewardship committee and thus requirement for consent was waived.

## Results

**Figure 1.**
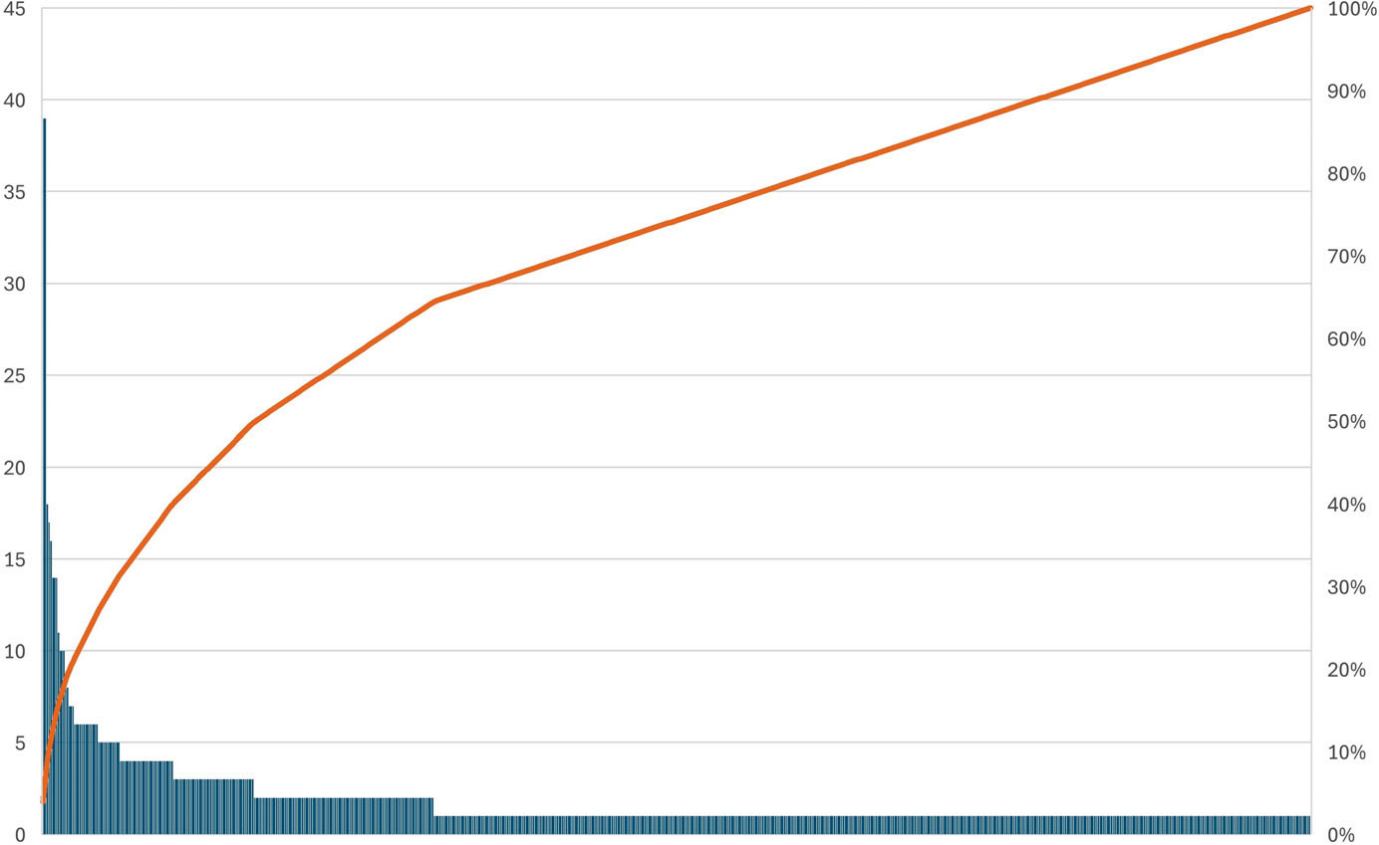
Pareto graph of clindamycin prescriptions at a large military healthcare system in 2023 by individual prescribers, from most to least prolific.

During the study period, data was collected from 1046 filled clindamycin prescriptions among 972 patients by 491 clinicians (Table 1). Patients were 44.9% male with a median age of 48 [interquartile range 27.5–66]. The median duration of therapy for clindamycin prescriptions was 7 days [IQR 7–10]. Most (58.5%) prescriptions were written for patients without a documented beta-lactam allergy. A minority (23.4%) of prescriptions were prescribed from outside the military health system. There was a total of 347 clinicians on the military base and 186 clinicians off base that prescribed clindamycin during the study period. While there were 533 distinct prescribers, ten (1.9%) had ten or more prescriptions for clindamycin during the study period; they accounted for 18.1% of all prescriptions (**Figure 1)**. These prescribers were in the emergency department, surgical sub-specialties, and primary care, and only one was affiliated with a graduate medical education program. These providers included four physicians, five physician assistants, and one nurse practitioner. Seven of these clinicians were civilian employees of the military and three were active duty. Of prescriptions from these providers, 68.8% were in the absence of a known beta-lactam allergy.

**Table 1.** Characteristics of the 1 046 prescriptions for outpatient clindamycin prescription at a large military healthcare system in 2023

Male (n, %)	470 (44.9%)
Age of patients, median [IQR]	48 [27.5–66]
Pediatric (Age <18)	76 (7.3%)
Adult (Age 18+)	970 (92.7%)
Active Duty	187 (17.9%)
Guard	18 (1.7%)
Reserve	17 (1.6%)
Days of therapy	7 [7–10]
Beta-lactam allergy	434 (41.5%)
Any antibiotic allergy	530 (50.7%)
Prescriptions from outside military health system	245 (23.4%)

Among clindamycin prescriptions within the military health system, 45.4% were prescribed by emergency medicine clinicians and 14.9% from surgery and surgical subspecialties (Table 2). The most common surgical specialties were plastic surgery (4.9%), otolaryngology (2.9%), and orthopedics (2.4%). While only 8% of prescriptions came from military dental providers, 56.3% of prescriptions from outside the healthcare system came from dental providers. Only two (0.2%) prescriptions were prescribed by infectious diseases.

**Table 2.** Prescribing clinics for outpatient clindamycin prescriptions at a large military healthcare system in 2023

Clinic Type	Prescriptions from inside the military health system (*n* = 801)	Prescriptions from outside the military health system (*n* = 245)
Emergency medicine	364 (45.4%)	11 (4.5%)
Dental/Oral surgery	64 (8.0%)	138 (56.3%)
Primary care and medical sub-specialties	189 (23.6%)	64 (26.1%)
Burn	43 (5.4%)	0 (0.0%)
Surgery and Surgical subspecialties	119 (14.9%)	29 (11.8%)
Obstetrics and gynecology	22 (2.7%)	3 (1.2%)

When comparing by diagnostic indication, there were a greater proportion of prescriptions in the setting of beta-lactam allergy for dental (*p* < 0.0001), pharyngitis/throat (*p* = 0.03), and postoperative (*p* < 0.0001) indications, where there were significantly more prescriptions without a beta-lactam allergy for skin and soft tissue infection (*p* < 0.0001), gynecologic infection (*p* = 0.01), and unknown (*p* = 0.01) indications (Table 3).

**Table 3.** Prescriptions by diagnostic group and beta-lactam allergy status of patient

Diagnostic Group	With beta-lactam allergy (*n* = 434)	Without beta-lactam allergy (*n* = 612)	*P–*value
Dental	167 (38.5%)	98 (16.0%)	<0.001
Skin and soft tissue infection	101 (23.3%)	314 (51.3%)	<0.001
Pharyngitis	53 (12.2%)	46 (7.5%)	0.01
Gynecological infections	4 (0.9%)	22 (3.6%)	0.01
Preoperative prophylaxis	4 (0.9%)	3 (0.5%)	0.40
Postoperative prophylaxis	43 (9.9%)	13 (2.1%)	<0.001
Sinusitis	8 (1.8%)	7 (1.1%)	0.35
Mastitis	1 (0.2%)	3 (0.5%)	0.50
Burns	1 (0.2%)	4 (0.9%)	0.33
Other	18 (4.1%)	37 (6.0%)	0.18
Unknown	34 (7.8%)	65 (10.6%)	0.13

## Discussion

In this retrospective study of one year’s worth of outpatient clindamycin prescriptions across a large military healthcare network with high levels of clindamycin resistance, we found several themes regarding the use of clindamycin. Firstly, we found that approximately half of the prescriptions were written in the setting of antibiotic allergy with a majority being beta-lactam allergies. Secondly, a few prolific prescribers accounted for almost a fifth of oral clindamycin prescriptions. Finally, we found that the specific indication for antimicrobials varied by presence of beta-lactam allergy.

The ideal way to decrease specific antimicrobials is challenging and multidisciplinary. Stewardship teams have access to tools such as prospective audit and feedback and restricted formularies. While many initiatives have focused on the inpatient use of clindamycin, there is a lack of data regarding strategies to reduce outpatient use.^10^ High clindamycin prescription rates in and of themselves may be an indicator of poor overall stewardship practices. For example, increase in the use of outpatient clindamycin, along with macrolides and fluoroquinolones, at rates higher than predefined use thresholds was associated with higher community prevalence density of MRSA.^11^ With its use both inpatient and outpatient, our study demonstrates the need to focus on both settings to decrease unnecessary clindamycin usage.

Many medical systems have struggled to increase adherence to recommendations.^12^ In this study, outpatient CPGs were produced to promote narrow spectrum antimicrobial use. Unfortunately, there is long-standing research suggesting that awareness of guidelines alone may not be enough to change practices.^13,14^ Dentists have been previously identified as common prescribers of clindamycin in the US and are frequently not included in traditional stewardship initiatives.^15^ For instance, clindamycin accounted for more than 25% of antibiotic prescriptions by outpatient dentists in Germany, which a recent study noted was likely inadequate and inconsistent with recommendations.^16^ This occurs in the United States as well in prescriptions of dental infections by non-dentists. Of 2.2 million ER visits for dental conditions in the US From 2011–2015, antibiotics were prescribed in 1.4 million (65%); of these, almost one quarter were clindamycin.^17^ A challenge noted in our study is that most of the dental prescriptions originated from outside the military base. Efforts focused on improving dental prescribing should ensure limiting antibiotics to appropriate indications, choosing the correct agents.

As almost half of the patients had beta-lactam allergies, this study highlights the need for safe secondary agents to consider in patients with antibiotic allergies as well as linkage programs to de-labeling, as many reported penicillin allergies occur in low-risk patients for whom re-challenge to facilitate removal of labeled allergy could be pursued.^18^ Hospitalized patients with recorded penicillin allergies have been found to be more likely to be exposed to clindamycin and have longer hospital stays.^19^ Previous research has identified reported penicillin allergy as being associated with a higher risk of surgical site infection.^20–23^ In contrast, de-labeling studies of patients with reported penicillin allergies in emergency and inpatient settings were able to successfully de-label the vast majority, which decreases healthcare utilization and should be a target of future efforts.^24–27^ Adding on to this, our work suggests that non-traditional areas, such as dental clinics, should be considered for allergy de-labeling efforts.

In identifying prolific departments and prescribers, this study is likely to have a meaningful impact on local stewardship efforts. The current local clinical practice guideline at this health care system recommends trimethoprim/sulfamethoxazole or doxycycline as first line options in the setting of purulent cellulitis and does not mention fluroquinolones or clindamycin. Analysis of diagnostic indications has identified plastic surgery as a specialty for whom intervention may prove valuable, as there remains a significant amount of postoperative clindamycin use. Additionally, a significant number of prescriptions were provided for patients in burn follow-up clinic without noting a specific concern for infection. We believe that all these areas represent opportunities for stewardship intervention.

Our study has several limitations. Given the wide array of diagnostic indications included, we were unable to apply appropriateness criteria for clindamycin prescriptions, such as patients who failed other therapies. We were also unable to include prescriptions for clindamycin that were not filled. Additionally, we did not have access to records for encounters outside of the military health system, and thus have limited details regarding these prescriptions. The number of diagnoses as well as procedures performed on and off the military base could inform rates of antibiotic prescription, but unfortunately that data is unavailable through the methods used for this study. While the local CPGs recommend against empiric clindamycin, it is currently unclear how many providers are aware of the guidelines.

## Conclusion

We found that over half of clindamycin prescriptions at this large military healthcare system occurred in the absence of known beta-lactam allergies. The proportion of clindamycin prescriptions in patients with beta-lactam allergies was different by diagnostic indication. It is also of note that a disproportionate number of prescriptions could be attributed to a small number of high-volume prescribers, suggesting that careful targeting of educational interventions may prove more efficacious. These findings highlight clindamycin prescribing in a large military healthcare network and aid in identifying areas to target for future interventions to decrease clindamycin use.
